# The Differential Effects of Vitamin K Across Glycaemic Outcomes in Prediabetes and Type 2 Diabetes Mellitus

**DOI:** 10.3390/nu18020269

**Published:** 2026-01-14

**Authors:** Syeda Ruwaida Ahmed, Kabelo Mokgalaboni, Wendy N. Phoswa

**Affiliations:** Department of Life and Consumer Sciences, College of Agriculture and Environmental Sciences, University of South Africa, Florida Campus, Roodepoort 1710, South Africa

**Keywords:** vitamin K, hyperglycaemia, insulin resistance, pancreas

## Abstract

**Background:** Vitamin K has emerged as a promising regulator of glucose metabolism in preclinical studies. There is, however, scant evidence to support this promising potential in a clinical setting. **Aim:** The aim of this study was to confirm the effects of vitamin K supplementation on glycaemic parameters such as fasting blood glucose (FBG), fasting insulin (FI), glycated haemoglobin (HbA1c), insulin resistance (HOMA-IR), and homeostatic model of beta cell function (HOMA-β) across randomised controlled trials (RCTs). **Materials and Methods**: This meta-analysis used evidence from PubMed, Scopus, and manual screening. Only RCTs were considered for this meta-analysis of interventional studies. The Meta online tool was used to analyse data, with the results reported as either the mean or the standardised mean difference (SMD), alongside 95% confidence intervals (CI). **Results:** Only eight RCTs were found relevant and analysed; the age of those in the vitamin K group was 50.58 ± 6.91 years, and in the control group, it was 48.19 ± 5.41. The evidence showed a significant reduction in FBG, SMD = −0.22 (−0.39 to −0.05), HbA1c, MD = −1.00%, 95% CI (−1.92 to −0.07), and HOMA-IR, MD = −0.63, 95% CI (−1.20 to −0.06). However, no effect was observed on insulin (SMD = −0.39, 95% CI: −0.91 to 0.13, *p* = 0.15) and HOMA-β (MD = 6.56, 95% CI (−3.89 to 17.01), *p* = 0.2184. Low doses of vitamin K2 and vitamin K1 were associated with reduced HbA1c and HOMA-IR, respectively. An intervention of less than 12 weeks was associated with reduced HOMA-IR. **Conclusions:** This study showed a significant decrease in FBG, HbA1c, and HOMA-IR without affecting insulin or HOMA-β. Nevertheless, the limited number of trials with moderate quality warrants larger, longer-term RCTs with rigorous methodology and direct comparisons of vitamin K isoforms to better assess therapeutic potential.

## 1. Introduction

Type 2 diabetes mellitus (T2D) is a chronic metabolic disorder characterised by impaired insulin secretion and/or action, resulting in persistent hyperglycaemia [1]. This condition remains a major contributor to global morbidity and mortality, affecting over 500 million individuals globally, with an anticipated rise to 200 million by 2045 [2]. T2D accounted for 90% of all diabetes cases reported in 2021, and 44.7% of adults globally were unaware of their status, with the highest rate in Africa at 53.6%, thereby increasing its prevalence [3]. Individuals with T2D have an increased risk of developing cardiovascular diseases (CVDs) and related complications, further exacerbating the global burden and mortality associated with the condition [4].

Additionally, persistent hyperglycaemia contributes to progressive tissue damage in diabetes, thus warranting the need to manage T2D effectively [5]. Moreover, long-term uncontrolled hyperglycaemia is often associated with microvascular complications, such as diabetic nephropathy, diabetic retinopathy, diabetic neuropathy, and macrovascular complications, such as peripheral arterial disease [5,6]. Uncontrolled hyperglycaemia in T2D is associated with vitamin K deficiency, an essential micronutrient involved in glucose metabolism [7,8,9].

Common conventional pharmacological treatments, such as sulfonylureas, meglitinides, thiazolidinediones, and Glucagon-like peptide-1 (GLP-1) receptor agonists, effectively improve glycaemic control but are often associated with adverse effects [10]. These include hypoglycaemia, weight gain, fluid retention, gastrointestinal disturbances, and, in some cases, increased risks of cardiovascular events or cancer [10,11]. Due to these potential limitations, there is growing interest in medicinal plants and dietary supplements as complementary or alternative interventions. For instance, existing reports have shown the anti-hyperglycaemic effects of *Corchorus olitorius* and okra in diabetes and prediabetes by reducing fasting blood glucose (FBG); the impact on glycated haemoglobin (HbA1c) is limited [12,13]. Other previous studies support the use of medicinal plants and their active compounds, which have demonstrated promising effects in reducing HbA1c, FBG, and insulin resistance, often with minimal side effects [14,15,16].

Despite the potential benefits of medicinal plants and pharmacological agents mentioned above, the adverse effects and limitations of the aforementioned antidiabetic interventions have prompted growing interest in the use of vitamins as therapeutic supplements, given their safety profiles, affordability, and physiological compatibility. Among these, vitamin K has emerged as a supplement of interest, as it has been shown to enhance insulin sensitivity and control of glycaemic outcomes in T2D patients, possibly through regulation of vitamin K-dependent proteins such as osteocalcin, positively affecting the microbiota that influences glucose homeostasis and decreasing inflammation [9,17]. Vitamin K exists in two primary forms: vitamin K1 (phylloquinone), predominantly found in green leafy vegetables, and vitamin K2 (menaquinone), present in fermented foods and synthesised by the gut microbiota [18,19]. Beyond its roles in coagulation, bone metabolism, and inhibition of vascular calcification [20], it has been shown to have antihyperglycaemic effects in rodent models of diabetes [21,22].

However, evidence from clinical trials disputes these effects [17,23], suggesting that the translation from in vivo to clinical data remains limited. A previous meta-analysis found no effect of vitamin K on FBG, HbA1c, or insulin, except on HOMA-IR [24]. On the other hand, a recent meta-analysis reported the effects of vitamin K2 on insulin, HbA1c, and homeostatic model of insulin resistance (HOMA-IR); however, the study did not assess FBG or HOMA-β, thereby limiting conclusions about antihyperglycaemic status [25]. Moreover, this study included a few trials (6) across a wide range of conditions, including polycystic ovarian syndrome, osteopenia, and T2D, which may limit statistical power. Another meta-analysis found no effect of vitamin K on markers of hyperglycaemia [17]. These studies also fail to establish the subgroup analysis among those with diabetes and prediabetes. The prior meta-analyses fail to distinguish between different forms of vitamin K as antihyperglycaemic agents. Meanwhile, other trials suggest otherwise [26,27]. Hence, these contradictory findings and limitations warrant a large-scale quantitative analysis to confirm the effects of vitamin K supplementation on glycaemic control in patients with prediabetes or T2D. This meta-analysis, with detailed subgroup analyses, provides evidence from RCTs confirming whether vitamin K can ameliorate hyperglycaemia in prediabetes and T2D.

## 2. Methodology

This meta-analysis adhered to the Preferred Reporting Items for Systematic Reviews and Meta-Analyses (PRISMA) 2020 guidelines to ensure transparent, high-quality, and ethical reporting [28]. The protocol was designed before study initiation and registered in the PROSPERO database (CRD42020151667) [29]. However, this has been amended to include pre-diabetes. The PRISMA flow diagram presents the number of studies identified, screened, excluded, and included in the final meta-analysis. The PICOS framework was used to develop research questions and to define the article screening process (Table 1). The results are reported in accordance with the PRISMA checklist (Table S1).

### 2.1. Literature Search and Strategy

A broad literature search was conducted across PubMed and Scopus, including manual screening of studies (Table S2). The search was restricted to those published from the time of database inception until 15 August 2025, with updates as of 1 January 2026. To ensure that all relevant studies were retrieved, umbrella medical subject heading (MeSH) terms were used, including “Vitamin K” AND “diabetes”. The restrictions included a study design to capture only RCTs.

The inclusion criteria for this meta-analysis comprised randomised controlled trials (RCTs) conducted among adult patients diagnosed with type 2 diabetes mellitus (T2D) or prediabetes. Eligible studies were required to involve vitamin K interventions, either as phylloquinone (vitamin K1) or menaquinone (vitamin K2), or menadione (vitamin K3), or acetomenaphthone, and to report at least one of these parameters: HbA1c, FBG, insulin, HOMA-IR, or HOMA-β. Only studies published in the English language were considered for inclusion. In contrast, observational designs, including literature reviews, cross-sectional studies, and case reports, were excluded, along with studies focusing on patients with type 1 diabetes mellitus or gestational diabetes. Additionally, studies presenting minimal or incomplete baseline and outcome data were excluded to ensure adequate data quality and comparability across included studies. Animal models of T2D and prediabetes were also excluded from this meta-analysis.

### 2.2. Data Extraction and Quality Assessments

The data from the studies that met the PICOS criteria were extracted by independent researchers (SRA and KM). Any disagreement regarding extraction was resolved by inviting a third independent researcher (WNP). Among the important items extracted were the authors’ last names and year of publication; age and BMI in both groups; gender distributions; sample sizes in both groups; the exact condition in each trial; and outcome data such as FBG, insulin, HbA1c, HOMA-IR, and HOMA-β. The risk of bias (ROB) was assessed across five main domains using https://mcguinlu.shinyapps.io/robvis/ (accessed on 11 November 2025).

### 2.3. Data Synthesis and Analysis

For data reported as median and range, we estimated mean and SD using the method devised by Hozo et al. in 2005 [30]. From each trial, the change in mean was calculated as the difference between the final and baseline means. At the same time, the SD was estimated using the Cochrane handbook formula: SD = square root of (SD baseline squared plus SD final squared minus 2 times the correlation coefficient (R) multiplied by the product of SD baseline and SD final). The correlation coefficient (R = 0.5) was adopted from a previous report [31,32]. Data analysis was performed using an online software by Fekete and Team [33] available at https://metaanalysisonline.com/ (accessed on 1 October 2025). For data presentation, mean difference (MD) or standardised mean difference (SMD) alongside 95% confidence intervals (CIs) was used. The MD was used for the outcome reported in the same unit of measurement, whereas the outcomes reported in different units were reported as SMD. Heterogeneity was assessed using *I*-squared statistics; *I*^2^ values greater than 50% were considered moderate heterogeneity. Subgroup analysis was conducted for evidence that showed high heterogeneity. In case of high heterogeneity, the random-effect model meta-analysis was adopted, while the fixed-effect model was used for small to zero heterogeneity [31]. Sensitivity tests were performed to establish the robustness of the analysed effect size, as previously reported elsewhere [34]. Publication bias was evaluated by visual inspection of the funnel plot and Egger’s regression test, where a *p*-value less than 0.05 suggested potential bias when coupled with funnel plot symmetry [35,36]. For all the outcome measures, a *p*-value of <0.05 was considered statistically significant.

## 3. Results

### 3.1. Databases and Study Selection

**Figure 1:**
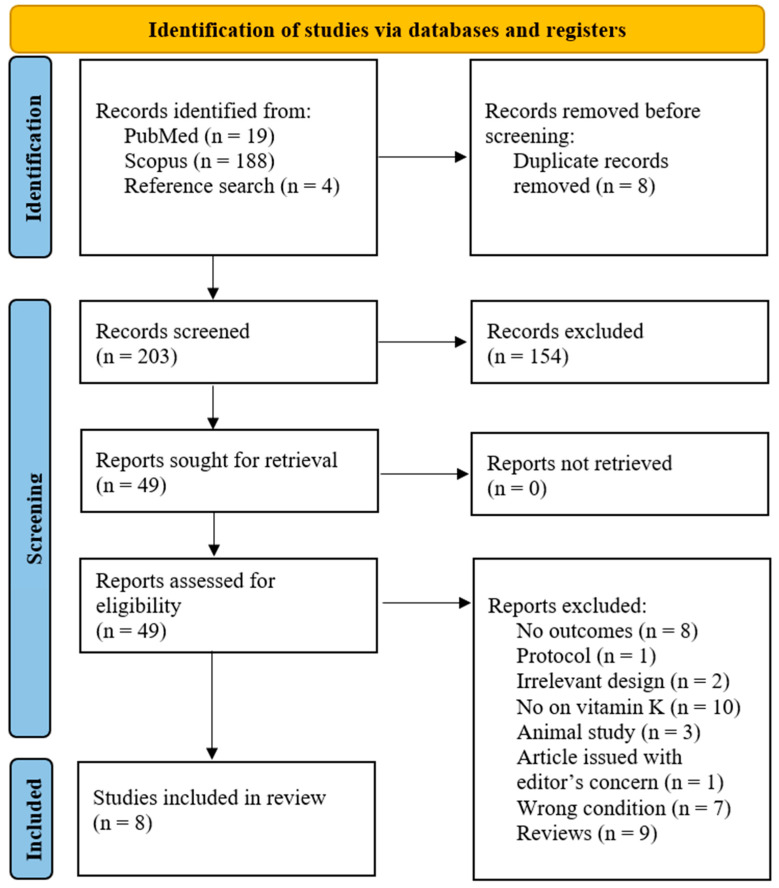
PRISMA flow diagram.

The screening procedure and inclusion criteria are illustrated in Figure 1. We identified 19 records in PubMed and 188 in Scopus, and an additional four studies were retrieved through a bibliographic search. After removing eight duplicates, the first screening of titles and abstracts resulted in the exclusion of 154 records, as they were not relevant to our focus. The full-text screening of the remaining 49 records resulted in the exclusion of 41 for various reasons. Among these included irrelevant study design, irrelevant treatment, animal models of diabetes, studies without relevant outcomes, irrelevant conditions, and studies that raised editors’ concerns, hence nullifying the findings drawn from their data. Only eight trials [26,27,37,38,39,40,41,42] met all the criteria outlined in our preplanned PICOS and were included in the meta-analysis.

### 3.2. Characteristics of Included Trials

**Figure 2:**
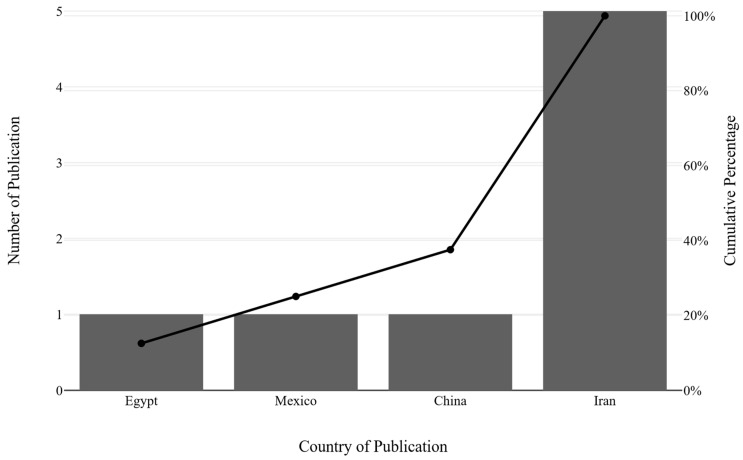
Distribution of trial publications over the years.

The current evidence included seven relevant trials [26,27,37,38,39,40,41,42] published between 2015 and 2023 (Table 2). The RCTs included a diverse population with at least 164 individuals with prediabetes and 306 with T2D. The trials were conducted in three countries (Figure 2). The age of patients in the vitamin K group was 50.58 ± 6.91 years. The baseline BMI in the vitamin K group was 28.94 ± 3.26 kg/m^2^. The dose of vitamin K used across all these trials ranged from a small (90 μg) to the highest dose (1000 μg). The duration of intervention also ranged from the shortest (4 weeks) to the longest duration of 26 weeks.

### 3.3. The Risk of Bias Across the Included Trials

**Figure 3:**
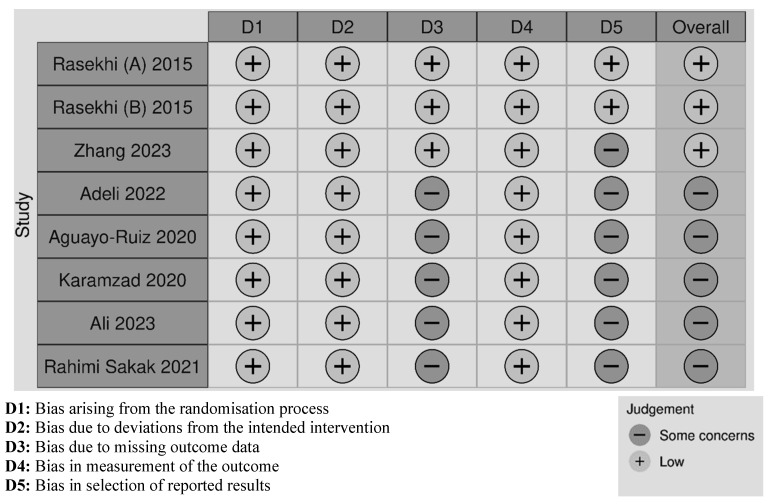
Cochrane risk of bias traffic light plot across the eight trials [26,27,37,38,39,40,41,42].

The risk of bias assessed across five domains is presented in Figure 3. The overall ROB ranged from low to high, with the majority classified as having some concerns due to bias associated with missing outcome data. The randomisation method, the intended intervention, and the measurement of outcome were sufficiently specified in all the trials.

### 3.4. Effects on Glycaemic Control

**Figure 4:**
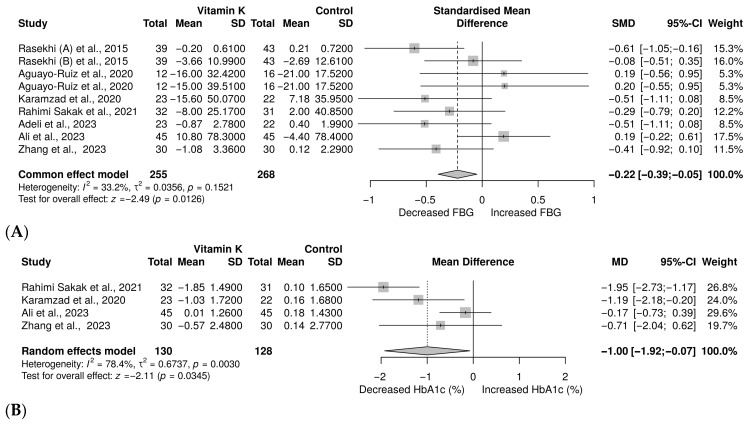
Forest plot showing the impact of vitamin K on glycaemic control: (**A**): FBG [26,27,37,38,39,40,41,42], (**B**): HbA1c [27,37,39,40].

The level of blood glucose was reported in eight trials with nine arms [26,27,37,38,39,40,41,42], involving 523 individuals with prediabetes and T2D. Meta-analysis demonstrated a small yet significant decrease in FBG, SMD = −0.22 (−0.39 to −0.05), *p* = 0.0126 (Figure 4A). Furthermore, there was a non-significant heterogeneity (*I*^2^ = 33.2%). HbA1c was reported in 4 trials [27,37,39,40] involving 258 patients with prediabetes or T2D. The random-effect model showed a significant reduction in HbA1c, MD = −1.00%, 95% CI (−1.92 to −0.07), *p* = 0.0345 (Figure 4B). However, significant heterogeneity was observed (*I*^2^ = 78.4%).

### 3.5. Effects on Insulin Resistance (HOMA-IR), Insulin Level, and HOMA-β Status

**Figure 5:**
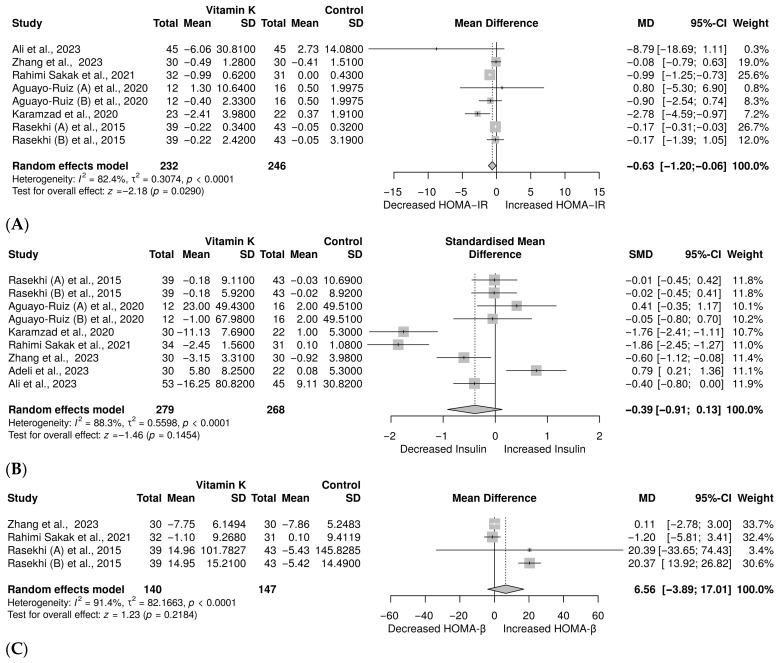
Meta-analysis on the effect of vitamin K on (**A**) HOMA-IR [27,37,38,39,40,41,42], (**B**) insulin [26,27,37,38,39,40,41,42], (**C**) HOMA-β [27,37,41,42].

HOMA-IR, a marker of insulin resistance, was measured in 7 with 8 [27,37,38,39,40,41,42] different treatment arms involving 478 individuals with prediabetes and T2D. The meta-analysis showed a significant decrease in HOMA-IR with vitamin K compared to control, MD = −0.63, 95% CI (−1.20 to −0.06), *p* = 0.0290 (Figure 5A). However, a substantial heterogeneity was observed (*I*^2^ = 82.4%). In contrast, eight trials with 9 [26,27,37,38,39,40,41,42] treatment arms with 547 patients with prediabetes and T2D were investigated to determine the effect on insulin. The results showed a decrease in insulin levels (SMD = −0.39, 95% CI: −0.91 to 0.13); however, this was not statistically significant (*p* = 0.1454) (Figure 5B). We also noted significant heterogeneity (*I*^2^ = 88.3%). In terms of HOMA-β, the evidence from four trials [27,37,41,42] with a sample size of 287 showed an increase in HOMA-β; however, this was not significant (MD = 6.56, 95% CI (−3.89 to 17.01), *p* = 0.2184; Figure 5C). Moreover, this evidence revealed significant heterogeneity (*I*^2^ = 91.4%).

### 3.6. Publication Bias

**Figure 6:**
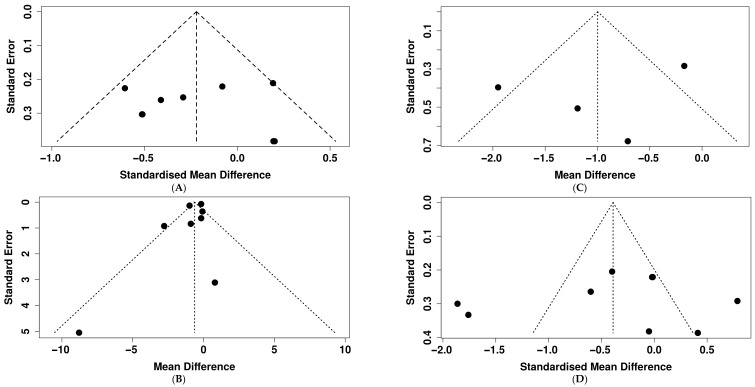
Assessment of publication bias through visual inspection of funnel plots across all outcomes: (**A**): FBG, (**B**): HbA1c, (**C**): HOMA-IR, (**D**): insulin.

For FBG, the funnel plot showed a symmetrical shape (Figure 6A). Consistent with this visual inspection, Egger’s regression test showed no evidence of small-study effects (*p* = 0.879). These findings suggest no evidence of publication bias in the included studies. For HbA1c, the funnel plot suggested some asymmetry (Figure 6B); however, Egger’s regression test did not indicate evidence of small-study effects (*p* = 0.56). Considering the limited number of trials and the low power of the asymmetric test, the observed funnel plot asymmetry may be due to between-study heterogeneity rather than publication bias. Similarly, for HOMA-IR, while the funnel plot suggested some asymmetry (Figure 5C), the Egger’s regression test did not indicate evidence of small-study effects (*p* = 0.332). Given the limited number of trials, these findings do not support the presence of publication bias. Although the funnel plot for insulin shows asymmetry (Figure 6D), the Egger’s test was not significant (*p* = 0.718). Due to high heterogeneity and few trials on this outcome, it is important to interpret the results with caution. For the HOMA-β, the visual inspection of the funnel plot does not show plot asymmetry (Supplementary Figure S1) as supported by the Eggers regression test (*p* = 0.654), suggesting no evidence of publication bias.

### 3.7. Subgroup Analysis

Subgroup analysis showed that vitamin K supplementation has dose-dependent metabolic effects (Table 3). A low dose of vitamin K (90–200 μg) significantly improved glycaemic control and insulin sensitivity, while a high dose (1000 μg) enhanced β-cell function. HbA1c reduction occurred in both groups; however, the low-dose group showed a more robust reduction. The high-dose of β-cell function was more reliable. The form of vitamin K also showed an effect on these metabolic parameters. Vitamin K2 significantly improved HbA1c and insulin resistance, while vitamin K4 was effective in enhancing β-cell function. Vitamin K1 consistently improved insulin resistance without effect on other markers. Patients aged 50 years or older showed significant improvement in glycaemic control and insulin resistance, whereas those aged 50 years or younger showed enhanced β-cell function. The glycaemic parameter differed with baseline BMI following vitamin K supplementation; for instance, overweight patients demonstrated greater improvement in HbA1c, while normal-weight patients showed reduced insulin levels; however, these findings were based on a single study. Vitamin K supplementation improved insulin resistance, with a greater effect noted after 12 weeks of intervention. Neither short nor long durations significantly improved β-cell function. In prediabetes, vitamin K improved β-cell function and insulin resistance, whereas in T2D, it improved insulin resistance without restoring resting β-cell function.

### 3.8. Sensitivity Analysis

For FBG, only the exclusion of Ali’s trial resulted in SMD = −0.31 (95% CI: −0.44 to −0.18), *p* = 0.0002, *I*^2^ = 4.93%. For HbA1c, removing Rahimi Sakak led to change effect size, MD = −0.57, 95% CI (−1.23 to 0.09), *p* = 0.09, *I*^2^ = 38.4%. Exclusion of Karamzad resulted in MD = −0.94, 95% CI (−2.18 to 0.30), *p* = 0.14, *I*^2^ = 85%. The exclusion of Ali resulted in an effect size of MD = −1.42 (95% CI: −2.13, −0.72), *p* < 0.001, *I*^2^ = 33.7%. Exclusion of the trial by Zhang led to MD = −1.08, 95% CI (−2.23 to 0.08), *p* = 0.07, *I*^2^ = 85.6%. According to the leave-one-out test, excluding both Rahimi Sakak and Ali contributed to the observed heterogeneity and effect size. For insulin, only excluding the Rahimi Sakak trial resulted in an SMD of −0.21 (95% CI: −0.51 to 0.09), *p* = 0.373, *I*^2^ = 83.0%. For HOMA-β, removing the prediabetes trial conducted by Rasekhi (B) changed the effect size to MD = −0.75, 95% CI (−4.20 to 2.69), *p* = 0.67, *I*^2^ = 0%. Altogether, these changes suggest that the HOMA-β result was not robust, as excluding Raskhi (B) reversed the effect size and eliminated heterogeneity. However, removal of Rasekhi (A) led to MD = 6.31, 95% CI (−6.78 to 19.40), *p* = 0.34, *I*^2^ = 93.8%. The exclusion of Rahimi Sakak changed the effect size to MD = 11.19, 95% CI (−7.10 to 29.47), *p* = 0.23, *I*^2^ = 89.3%. Zhang, MD = 10.55, 95% CI (−9.10 to 30.20), *p* = 0.29, *I*^2^ = 93.6%.

## 4. Discussion

This study reviewed clinical trials that examined the antihyperglycaemic effects of vitamin K supplementation in individuals with T2D and pre-diabetes. The pooled effect size indicated a modest, significant decrease in FBG following vitamin K supplementation in both prediabetics and T2D. Additionally, vitamin K demonstrated a large, clinically significant reduction in HbA1c and a moderate improvement in insulin resistance. However, no effect was observed on insulin and HOMA-β. These suggest that improvement in insulin sensitivity may occur irrespective of changes in circulating insulin levels. Given that insulin resistance alongside pancreatic β-cell failure are the central features of T2DM [43], the data from this study suggest that vitamin K may prevent premature metabolic imbalance associated with insulin resistance, especially in patients living with prediabetes and T2DM. Our finding on HOMA-IR is consistent with the report by Zhao et al. [24]. Similarly, our results are partially supported by previous work showing significant benefits of vitamin K on HbA1c, HOMA-IR, and HOMA-β despite null findings on FBG [44]. On the other hand, the findings that are completely contrary to those reported here were reported in a previous meta-analysis, which showed no effect of vitamin K on insulin sensitivity [17]. Another meta-analysis also showed no effect on glycaemic control and insulin resistance in prediabetes and healthy individuals [23]. These findings might be attributable to the small sample size and the inclusion of a wide range of conditions, among other factors. While our findings showed an observational non-significant increase in HOMA-β, this contradicts findings from a rodent model of diabetes, which demonstrated an improvement in pancreatic β-cell function following vitamin K2 [21]. Based on our subgroup analysis, prediabetes could benefit more from vitamin K in reducing insulin resistance and improving β-cell function, as a pronounced effect was observed compared to T2D.

The observed vitamin K effect is supported by its ability to modulate multiple biochemical pathways. It primarily acts as a cofactor in the γ-carboxylation of undercarboxylated osteocalcin, which increases β-cell proliferation, promotes insulin secretion, and improves insulin sensitivity in peripheral tissues [45,46,47]. Moreover, vitamin K activates Matrix Gla Protein (MGP), which protects vascular and metabolic tissue from calcification and oxidative stress, thus increasing glucose uptake [48]. Evidence from rodents suggests that vitamin K2 may improve mitochondrial function, ATP production, and reduce reactive oxygen species (ROS), thereby enhancing insulin receptor signalling [49,50]. In diabetes, nuclear factor kappa beta (NF-κβ) is activated, resulting in upregulation of interleukin-6 (IL-6) and tumour necrosis factor alpha (TNF-α) [51]. These inflammatory cytokines interfere with the insulin signalling pathway by phosphorylating insulin receptor substrate 1 (IRS-1). This activity suppresses Phosphoinositide 3-kinase/protein kinase B (PI3K/Akt) signalling, reducing glucose transporter 4 (GLUT4) translocation and glucose uptake in muscle and adipose tissues [52,53]. The role of vitamin K in the inhibition of NF-κβ activation reduces IL-6 and TNF-α production, thus promoting the normal function of IRS-1 and insulin receptors [54,55,56]. Altogether, these activities reduce FBG and HbA1c and improve HOMA-IR. An overexpression of inducible nitric oxide synthase (iNOS) in T2D has been described by other researchers as a cause of hyperglycaemia [57,58]. Excess iNOS promotes the production of nitric oxide (NO), which then reacts with peroxynitrite, impairing insulin receptor signalling and, subsequently, damaging β-cell function [59,60]. An increased NO reduces mitochondrial efficiency, limiting ATP production required for insulin secretion [61]. Therefore, the inhibition of iNOS by vitamin K reduces oxidative stress, insulin receptor signalling, and β-cell function, thereby increasing glucose uptake [55]. While vitamin K presents potential benefits, in T2D, it should be used only when prescribed, as T2D patients are often placed on warfarin to manage CVD comorbidities. This can interfere with the efficacy of vitamin K and lead to severe hypoglycaemia [62].

Several strengths and limitations worth noting were encountered during this meta-analysis. A primary strength of this study is the inclusion of RCTs sourced from different databases. Additionally, significant heterogeneity across outcomes was observed, which may affect the overall interpretation of the results. We, however, conducted a subgroup analysis to find the source of variation. There were differences in the forms of vitamin K used (K1, K2, K3, and K4), dosages, mode of delivery, and duration of intervention. One notable limitation was the sample size: only eight trials were included in the current analysis, which reduced overall statistical power. These further limit subsequent tests and the reliability of other tests, including the subgroup analysis and the interpretation of funnel plots and Egger regression tests. However, interpreting publication bias using the funnel plot and Egger’s test is limited by small sample sizes. We found no evidence of bias across all hyperglycaemia outcome measures. Due to the limited sample size, meta-regression was not possible, as an underpowered sample could have led to false-positive or false-negative results. We also noted a geographic concentration of publications, with 71% of trials conducted in Iran, a country with a high prevalence of diabetes. The quality of included trials varied, with 37.5% high-quality and 62.5% moderate. For moderate-quality trials with some concerns, ROB trials seem to overestimate effect sizes and confidence intervals. Although knowledge of vitamin K status in these patients is essential for understanding the overall effect of vitamin K supplements, in the analysis, not all trials reported this, which limits our interpretation. All these key factors may affect the overall potential of the findings. Therefore, caution must be taken when interpreting these findings. Future research should focus on RCTs with larger sample sizes; rigorous methodology, including longer supplementation periods; and comparisons of the comparative efficacy of vitamin K forms. Future trials should also assess the baseline vitamin K status in the patients before supplements are given.

## 5. Conclusions

The data reported in this meta-analysis indicate that vitamin K supplementation may reduce FBG, HbA1c, and HOMA-IR without impact on insulin or HOMA-β in individuals with prediabetes and T2D. Although vitamin K cannot replace standard antidiabetic medications, the results support its role as a complementary approach to metabolic regulation, particularly for reducing hyperglycaemic markers. Given the high number of moderate-quality trials included in this study, future large trials with rigorous methodology are needed to determine the optimal dosing, treatment duration, and comparative efficacy of different vitamin K isoforms in diabetes.

## Figures and Tables

**Table 1:** PICOS criteria for the meta-analysis.

PICOS Element	Description
Population (P)	Adults with prediabetes and type 2 diabetes mellitus
Intervention (I)	Any form of vitamin K (phylloquinone, menaquinone, or menadione)
Comparison (C)	Placebo or other antidiabetic treatments
Outcome (O)	Glycaemic markers (HbA1c, FBG, HOMA-IR, HOMA-β or insulin)
Study Design (S)	RCTs

HbA1c: glycated haemoglobin; FBG: fasting blood glucose; HOMA-IR: homeostatic model assessment of insulin resistance; HOMA-β: homeostatic model of beta cell function; RCTs: randomised controlled trials.

**Table 2:** Basic characteristics of the nine studies published between 2015 and 2023.

Study, Year	Study Design (S)	Country	Population	Intervention Dose and Duration	Baseline Age (Year) Vitamin K Group	Baseline BMI (kg/m^2^) in Vitamin K Group	Gender (m/f) in Vitamin K Group	Control	Outcomes
Adeli et al., 2023 [26]	RCT	Iran	60 T2D	200 µg/d of MK-7 capsules for 12 weeks	45.35 ± 6.25	30.30 ± 2.23	16/7	Placebo	FPG and FI
Ali et al., 2023 [40]	RCT	Egypt	90 T2D	1 mg (1000 μg) of vitamin K4 for 24 weeks	49.68 ± 8.26	34.60 ± 5.33	5/40	Placebo	HbA1c, FPG, HOMA-IR, and FI
Zhang et al., 2023 [27]	RCT	China	60 T2D	90 µg of MK-7 in 100 g of yoghurt for 6 months	63.33 ± 1.33	24.98 ± 0.54	13/17	Placebo (yoghurt)	HbA1c, FPG, HOMA-IR, HOMA-β, and FI
Rahimi Sakak et al., 2021 [37]	RCT	Iran	68 T2D	180 µg vitamin K2 capsules twice a day for 12 weeks	58.50 ± 7.24	27.50 ± 3.69	14/20	Placebo	HbA1c, FPG, HOMA-IR, HOMA-β, and FI
Aguayo-Ruiz (A) et al., 2020 [38]	RCT	Mexico	28 T2D	100 μg of vitamin K2 plus calcinated magnesium for 3 months	55.42 ± 12.62	28.49 ± 7.44	NS	Vitamin D3 plus calcinated magnesium	HOMA-IR, and FI
Aguayo-Ruiz (B) et al., 2020 [38]	RCT	Mexico	28 T2D	100 μg vitamin K2 plus 1000 IU vitamin D for 3 months	57.08 ± 9.59	27.91 ± 4.55	NS	Vitamin D3 plus calcinated magnesium	HOMA-IR, and FI
Karamzad et al., 2020 [39]	RCT	Iran	60 T2D	200 µg/d MK-7 for 12 weeks	45.35 ± 6.25	30.30 ± 2.23	16/7	Placebo	HbA1c, FPG, HOMA-IR, and FI
Rasekhi (A) et al., 2015 [41]	RCT	Iran	82 prediabetes	One capsule containing 1000 μg of PK for 4 weeks	40.25 ± 5.32	28.34 ± 1.72	0/39	Placebo	FPG, HOMA-IR, HOMA-β, and FI
Rasekhi (B) et al., 2015 [42]	RCT	Iran	82 prediabetes	One capsule containing 1000 µg of PK for 4 weeks	40.25 ± 5.32	28.08 ± 1.65	0/39	Placebo	FPG, HOMA-β, and FI

RCT: randomised controlled trials; HOMA-IR: homeostasis model of assessment insulin resistance; HOMA-β: homeostatic model of beta cell function; HbA1c: glycated haemoglobin; FBG: fasting blood glucose; FI: fasting insulin; T2D: type 2 diabetes mellitus; MK-7: menaqionone-7; PK: phylloquinone; NS: not specified; m: male; f: female.

**Table 3:** Subgroup analysis for outcome with substantial heterogeneity.

Variable	Number of Studies	Group	Class	MD, 95% CI	*I* ^2^
HbA1c	3	Dose	Low	−1.42, (−2.13, −0.72) *	33.7
	1		High	−1.00 (−1.92, −0.07) *	N/A
HOMA-β	2	Dose	Low	−0.84 (−4.30, 2.60)	0
	2		High	20.37 (13.97, 26.77) *	0
HOMA-IR	3	Dose	Low	−0.89 (−1.63, −0.14) *	60.1
	5		High	−0.22 (−0.94, 0.51)	31.3
Insulin	6	Dose	Low	−0.52 (−1.42, 0.38)	91.8
	3		High	−0.16 (−0.41, 0.10)	8.7
HbA1c	3	Form	VK2	−1.42 (−2.13, −0.72) *	33.7
	1		VK4	−0.17 (−0.73, 0.39)	N/A
HOMA-β	2	Form	VK2	−0.84 (−4.30, 2.62)	0
	2		VK4	20.37 (13,97, 26.77) *	0
HOMA-IR	5	Form	VK2	−0.89 (−1.63, −0.14) *	60.1
	1		VK4	−8.79 (−18.69, 1.11)	N/A
	2		VK1	−0.17 (−0.31, −0.03)	0
Insulin	6	Form	VK2	−0.52 (−1.42, 0.38)	91.8
	1		VK4	−0.40 (−0.80, 0.00)	N/A
	2		VK1	−0.02 (−0.32, 0.29)	0
HbA1c	2	Age	Over 50 years	−1.45 (−2.64, −0.26) *	59.8
	2		Under 50 years	−0.59 (−1.58, 0.39)	67.5
HOMA-β	2	Age	Over 50 years	−0.84 (−4.30, 2.62)	0
	2		Under 50 years	20.37 (13.97, 26.77) *	0
HOMA-IR	4	Age	Over 50 years	−0.65 (−1.29, −0.02) *	48.8
	4		Under 50 years	−0.94 (−2.26, 0.38)	72.3
Insulin	4	Age	Over 50 years	−0.55 (−1.50, 0.40)	88.6
	5		Under 50 years	−0.26 (−0.91, 0.13)	88.8
HbA1c	1	BMI	Overweight	−1.95 (−2.73, −1.17)	N/A
	2		Obesity	−0.59 (−1.58, 0.39)	67.5
	1		Normal weight	−0.71 (−2.04, 0.62)	N/A
HOMA-β	3	BMI	Overweight	10.55 (−9.10, 30.20)	93.6
	1		Normal weight	0.11 (−6.49, 6.71)	N/A
HOMA-IR	5	BMI	Overweight	−0.53 (−1.16, 0.10)	86.4
	2		Obesity	−3.73 (−8.04, 0.57)	27
	1		Normal weight	−0.08 (−0.79, 0.63)	N/A
Insulin	5	BMI	Overweight	−0.32 (−1.05, 0.42)	88.3
	3		Obesity	−0.45 (−1.71, 0.81)	94
	1		Normal weight	−0.60 (−1.12, −0.08)	N/A
HOMA-β	1	Duration	<12 weeks	0.11 (−6.49, 6.71)	N/A
	3		≥12 weeks	10.55 (−9.10, 30.10)	93.6
HOMA-IR	2	Duration	<12 weeks	−0.17 (−0.31, −0.03) *	0
	6		≥12 weeks	−0.95 (−1.75, −0.15)	59.9
Insulin	2	Duration	<12 weeks	−0.02 (−0.32, 0.29)	0
	7		≥12 weeks	−0.50 (−1.21, 0.20)	90.3
HOMA-β	2	Condition	T2D	−0.84 (−4.30, 2.62)	0
	2		Prediabetes	20.37 (13.97, 26.77) *	0
HOMA-IR	6	Condition	T2D	0.95 (−1.75, −0.15) *	59.9
	2		Prediabetes	−0.17 (−0.31, −0.03) *	0
Insulin	7	Condition	T2D	−0.50 (−1.21, 0.20)	90.3
	2		Prediabetes	−0.02 (−0.32, 0.29)	0

HOMA-IR: homeostasis model of assessment insulin resistance; HOMA-β: homeostatic model assessment of β-cell function; HbA1c: glycated haemoglobin; T2D: type 2 diabetes mellitus, NA: not applicable, * statistically significant results.

## Data Availability

No new data was created in this study. Data sharing is not applicable to this article.

## Supplementary Materials

The following supporting information can be downloaded at https://www.mdpi.com/article/10.3390/nu18020269/s1. Table S1: PRISMA checklist, Table S2: Literature search on databases, Figure S1: Publication bias for HOMA-β.
