# Supplementary material for: The Differential Effects of Vitamin K Across Glycaemic Outcomes in Prediabetes and Type 2 Diabetes Mellitus
Source: Nutrients. 2026 Jan 14;18(2):269. doi: 10.3390/nu18020269 (PMC12845305; doi:10.3390/nu18020269)
Supplement: Supplementary file 1 [file nutrients-18-00269-s001.zip › nutrients-4070069-supplementary/Table S2. Literature search on databases.pdf]

Table S2: Literature search on databases

Database/	MesH Terms	Exact search	Number	Restrictions	Final search	Number
PubMed	Vitamin K<br>Vitamin K1<br>Phylloquinone<br>Vitamin K2<br>Menaquinone<br>Menadione<br>Diabetes	((((((vitamin K[MeSH Terms]) OR (vitamin K1[MeSH Terms])) OR (phylloquinone[MeSH Terms])) OR (vitamin K2[MeSH Terms])) OR (menaquinone[MeSH Terms])) OR (menadione[MeSH Terms])) AND (diabetes[MeSH Terms])	162	RCTs<br>English	((((((vitamin K[MeSH Terms]) OR (vitamin K1[MeSH Terms])) OR (phylloquinone[MeSH Terms])) OR (vitamin K2[MeSH Terms])) OR (menaquinone[MeSH Terms])) OR (menadione[MeSH Terms])) AND (diabetes[MeSH Terms]) Filters: Randomized Controlled Trial, English	19
Scopus	Vitamin K<br>Diabetes<br>Randomised controlled trials	(TITLE-ABS-KEY (vitamin k) OR TITLE-ABS-KEY (vitamin K1) OR TITLE-ABS-KEY (phylloquinone) OR TITLE-ABS-KEY (vitamin K2) OR TITLE-ABS-KEY (menaquinone) OR TITLE-ABS-KEY (menadione) AND TITLE-ABS-KEY (diabetes) AND TITLE-ABS-KEY (randomised controlled trials))	314	Article,<br>English	(TITLE-ABS-KEY (vitamin k) OR TITLE-ABS-KEY (vitamin K1) OR TITLE-ABS-KEY (phylloquinone) OR TITLE-ABS-KEY (vitamin K2) OR TITLE-ABS-KEY (menaquinone) OR TITLE-ABS-KEY (menadione) AND TITLE-ABS-KEY (diabetes) AND TITLE-ABS-KEY (randomised controlled trials)) AND (LIMIT-TO (DOCTYPE , "ar")) AND (LIMIT-TO (LANGUAGE , "English"))	188
