# Supplementary figures and images for: The Differential Effects of Vitamin K Across Glycaemic Outcomes in Prediabetes and Type 2 Diabetes Mellitus
Source: Nutrients. 2026 Jan 14;18(2):269. doi: 10.3390/nu18020269 (PMC12845305; doi:10.3390/nu18020269)

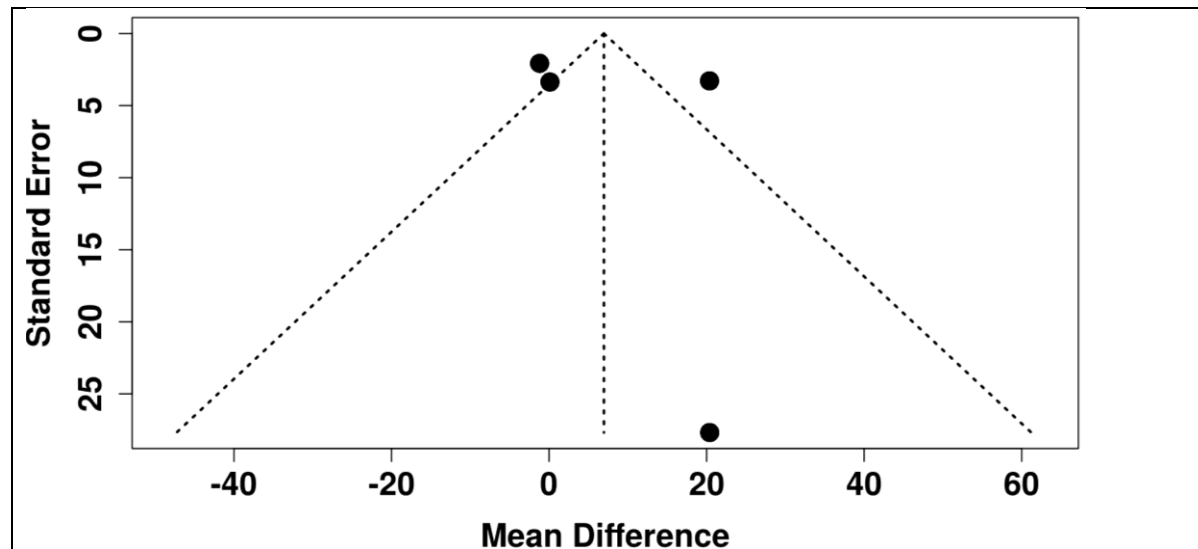

Figure 1: Publication bias for HOMA- $\beta$

Supplement: Supplementary file 1 [file nutrients-18-00269-s001.zip › nutrients-4070069-supplementary/Figure S1. Publication bias for HOMA-β.pdf]
